# Increased Adaptation Rates and Reduction in Trial-by-Trial Variability in Subjects with Cerebral Palsy Following a Multi-session Locomotor Adaptation Training

**DOI:** 10.3389/fnhum.2016.00203

**Published:** 2016-05-04

**Authors:** Firas Mawase, Simona Bar-Haim, Katherin Joubran, Lihi Rubin, Amir Karniel, Lior Shmuelof

**Affiliations:** ^1^Department of Biomedical Engineering, Ben-Gurion University of the NegevBeer-Sheva, Israel; ^2^Department of Physical Medicine and Rehabilitation, Johns Hopkins School of Medicine, BaltimoreMD, USA; ^3^Zlotowski Center for NeuroscienceBeer-Sheva, Israel; ^4^Department of Physical Therapy, Ben-Gurion University of the NegevBeer-Sheva, Israel; ^5^Department of Brain and Cognitive Sciences, Ben-Gurion University of the NegevBeer-Sheva, Israel; ^6^Department of Physiology and Cell Biology, Ben-Gurion University of the NegevBeer-Sheva, Israel

**Keywords:** adaptation, execution variability, rehabilitation, asymptotic performance, gait

## Abstract

Cerebral Palsy (CP) results from an insult to the developing brain and is associated with deficits in locomotor and manual skills and in sensorimotor adaptation. We hypothesized that the poor sensorimotor adaptation in persons with CP is related to their high execution variability and does not reflect a general impairment in adaptation learning. We studied the interaction between performance variability and adaptation deficits using a multi-session locomotor adaptation design in persons with CP. Six adolescents with diplegic CP were exposed, during a period of 15 weeks, to a repeated split-belt treadmill perturbation spread over 30 sessions and were tested again 6 months after the end of training. Compared to age-matched healthy controls, subjects with CP showed poor adaptation and high execution variability in the first exposure to the perturbation. Following training they showed marked reduction in execution variability and an increase in learning rates. The reduction in variability and the improvement in adaptation were highly correlated in the CP group and were retained 6 months after training. Interestingly, despite reducing their variability in the washout phase, subjects with CP did not improve learning rates during washout phases that were introduced only four times during the experiment. Our results suggest that locomotor adaptation in subjects with CP is related to their execution variability. Nevertheless, while variability reduction is generalized to other locomotor contexts, the development of savings requires both reduction in execution variability and multiple exposures to the perturbation.

## Introduction

Despite the rhythmic and automatic nature of locomotion, our walking pattern continuously adapts to changes in the environment and in our bodies (Gordon et al., 1995; Reisman et al., 2005). In the laboratory, locomotor adaptation can be studied using a split-belt treadmill that imposes different walking speeds to each leg. It has been shown that when subjects are exposed to such a perturbation, they change the velocity of the right and left strides to maintain the stability and the efficiency of their walking pattern (Reisman et al., 2005; Choi et al., 2009). Such independent control of spatial and temporal walking parameters to maintain symmetricity (Malone et al., 2011; Malone and Bastian, 2014; Roemmich and Bastian, 2015) can also be seen in turns (Weber et al., 1998). Locomotor adaptation requires detecting a deviation from symmetricity and correcting for that deviation, which both depend on the quality of the performed movements and of the sensory input (Miall and Wolpert, 1996). Addressing the interaction between execution and learning abilities is important for improving the diagnosis and the proposed treatments for patients that have movement disorders.

Cerebral Palsy (CP), caused by damage to motor control areas during development, is an umbrella term for a variety of movement disorders affecting movement and posture, and characterized by global deficits in locomotor and manual skills (Jahnsen et al., 2004). In addition to their deficits in movement execution, people with CP also show impairments in motor planning and learning, including grip force scaling for novel objects (Duff and Gordon, 2003), inadequate coordination and timing of forces (Nashner et al., 1983; Crenna and Inverno, 1994; Forssberg, 1999), and deficits in anticipatory adjustments and in sensory integration (Eliasson et al., 1995; Valvano and Newell, 1998; Hadders-Algra et al., 1999; Brogren et al., 2001). This broad spectrum of motor impairments in CP clearly demonstrates that in addition to the classical reported deficits in execution, people with CP also show deficits in control and in adaptation to external perturbations.

Still, it is unclear whether execution and learning deficits in people with CP are the outcome of damage to the same control process or are the result of damage to dissociable processes. Furthermore, it is unclear whether and how reduction of execution variability through training affect the adaptation process, and specifically, how it would affect the learning rate of people with CP that typically suffer from poor locomotor skills. To investigate if adaptation deficits in people with CP are related to their increased execution variability, we exposed subjects with CP and control subjects to a multi-session locomotor adaptation training using a split-belt treadmill. We hypothesized that reduction in performance variability will be associated with improved adaptation to an external perturbation.

## Materials and Methods

### Subjects

Six persons with diplegic CP [one female, mean age 17.4 ± 2.6 (SD) years] and nine typically developing control subjects [four females, mean age 19.4 ± 0.5 (SD) years] were recruited for the study (**Table 1**). Ethical approval for this study was granted from the local ethical review board of Assaf-Harofeh Medical Center, Israel. Written informed consent to participate in the study was obtained from participants or their parents/guardians after a detailed explanation of the study. Since CP is clinically heterogeneous, we restricted our recruitment to subjects with a moderate motor dysfunction of levels II and III according to the Gross Motor Function Classification System (GMFCS; Wood and Rosenbaum, 2000). Subjects in level III need assistive mobility devices and frequently use orthoses during walking, whereas subjects in level II do not need mobility devices and are frequently able to walk independently. Subjects with GMFCS II walked independently while subjects with GMFCS III held the side bars sporadically. Subjects that use an ankle-foot orthotic wore it during the experiment as well. The inclusion criteria for the CP group were: diagnosis of bilateral diplegia spastic CP; age from 12 to 22 years; GMFCS at level II or III, classified with their usual shoes, orthotics, and assistive mobility devices; cognitive level sufficient to comprehend and cooperate with measurements; and no orthopedic surgery or other spasticity management within the previous 6 months. Participants were excluded if they showed additional neurological symptoms, such as indicating a damage to the cerebellum or orthopedic conditions affecting the legs or back.

**Table 1:** Subject characteristics

	Gender (Male/Female)	Age (years, months)	BMI	GMFCS	Walking aids
Subjects					
S1	M	16 years, 7 months	22.3	III	Crutches
S2	M	19 years, 11 months	22.7	II	None
S3	F	19 years, 7 months	22.9	II	None
S4	M	19 years, 7 months	23	II	None
S5	M	16 years, 2 months	20.3	III	Crutches
S6	M	12 years, 8 months	15.2	II	None

mean ± (SD)		17.4 ± 2.6	21.1 ± 2.7		

*BMI, body mass index; GMFCS, gross motor function classification system; SD, standard deviation.*

**FIGURE 1:**
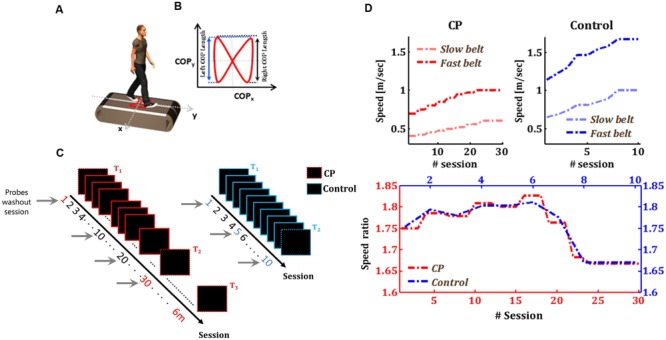
**Experimental design and protocols. (A)** Subjects walked on a split-belt force treadmill with two separate belts with an embedded force plate (white plate). The red trace represents the center of pressure (COP) profile for one gait cycle. **(B)** Schematic example for one COP profile for one cycle. **(C)** Training protocol for CP (red rectangles) and control group (blue rectangles). *T*_1_ and *T*_2_ represent the two test time-points in both groups in which subjects performed *the Baseline-Adaptation-Washout* paradigm. In the rest of the sessions, subjects were exposed only to the speed perturbation induced by the split-belt treadmill. In *T*_3_, the CP group did an additional test session, performed 6 months after the end of the adaptation sessions. Gray arrows represent the probes washout sessions. **(D)** Belt speed in CP and Control groups. Upper left panel: fast (red line) and Slow (light red) belt speeds across sessions in the CP group. Upper right panel: fast (blue line) and Slow (light blue) belt speeds across sessions in the Control group. Bottom panel: speed ratio across sessions in both groups (red and blue curves for CP and Control, respectively).

Subjects were instructed to walk on a custom split-belt force treadmill, which has two separate belts and an embedded force plate underneath (**Figure 1A**). The speed of each treadmill belt was controlled independently by its own motor. The belts could either be tied together and move at the same speed or split and move at different speeds. Subjects were instructed to walk without looking at their feet, to exclude any influence of visual feedback from the feet on the adaptation process. Safety harness, emergency stop buttons and adjustable sidebars were used for safety. Subjects were allowed to watch cartoons presented on a screen that was placed one meter away from the treadmill front. Although watching television programs and performing cognitive tasks during locomotor adaptation have been shown to reduce learning rates (Malone and Bastian, 2010), the fact that the cartoons were presented in all experimental sessions, and that subjects were not given any cognitive task with respect to the cartoons, suggests that this components will not interfere with the current experimental investigation.

### Protocol

Subjects with CP were exposed, during a period of approximately 4 months, to 30 sessions of split-belt adaptation (approximately two sessions per week). In each adaptation session, subjects walked with the belts of the split-belt treadmill moving at different speeds. In the first (i.e., *T*_1_) and last (i.e., *T*_2_) test sessions, subjects performed the *Baseline-Adaptation-Washout* protocol. Six months after the end of the adaptation sessions, subjects underwent an additional follow-up post-training test with the same *Baseline-Adaptation-Washout* test (i.e., *T*_3_). Age-matched healthy control subjects underwent ten sessions of adaptation to a split-belt treadmill with *Baseline-Adaptation-Washout* test sessions on the first and last days (i.e., *T*_1_, and *T*_2_, respectively), as shown in **Figure 1C**. The reason behind restricting the training of the control group for 10 session was the belief that control subjects will reach an asymptote in performance after less than 10 training sessions and since we were primarily interested in collecting data for the initial locomotor adaptation performance from age-matched controls. To estimate the generalization of the gains in learning and in execution variability to the non-perturbed condition, we assessed the adaptation of subjects to a washout period (tied-belts condition) in sessions 10 and 20 for CP group and in session 5 for the control group.

For each group, average speed values across the two belts were chosen to be within ±40% of comfortable self-pace walking speed (Palisano et al., 1997). Walking speeds increased gradually between sessions (**Figure 1D**); subjects began the training with a low speed (i.e., 0.6^∗^self-paced speed) in the first test session (i.e., *T*_1_), and ended the training at the upper limit of the speed (i.e., 1.4^∗^self-paced speed) in the last test session (i.e., *T*_2_). This gradual change of belt speeds across sessions was made in order to challenge and motivate subjects in their prolonged training. Averaged self-paced speeds was 0.7 and 1.1 m/s for CP and control group, respectively. The ratio of the speeds of the belts was equally maintained across groups (**Figure 1D**), where the fast leg walked 1.65–1.8 times faster than the slow one (**Figure 1D**).

During the *baseline* period, subjects walked with the two belts tied together at a slow speed (0.4 and 0.65 m/s for CP and control group in the first test session, respectively) for 1 min, then at a high speed (0.7 and 1.14 m/s for CP and control group, respectively) for another minute, and finally at low speed again for another minute. During the *adaptation* period, subjects walked for 15 min on a split-belt regimen where each leg was exposed to a different belt-speed. In the CP group, the subject’s more impaired leg walked on the faster belt, and the less impaired leg walked in the slower belt. Leg impairment for the subjects with CP was determined using functional tests conducted by professional physiotherapists. The decision to train the more impaired leg on the fast belt was made in order to exaggerate step length asymmetry during split-belt adaptation (Reisman et al., 2013). During the *washout* period, subjects walked with the two belts tied together at a slow speed for 5 min at the first session and for 10 min at the last treatment and washout sessions. The speed in the washout sessions was determine based on the speed of the slow-belt during the adaptation of the same session. In these washout sessions, subjects with CP walked with belts speed of 0.4, 0.47, 0.55, and 0.6 m/sec in sessions 1, 10, 20, and 30, respectively. Control subjects walked with belts speed of 0.65, 0.81, and 1 m/sec in sessions 1, 5, and 10, respectively. At the end of each period, the treadmill stopped for a 30 s break for resetting the treadmill’s belt speed. Subjects were not notified about the perturbation schedule.

### Data Collection

The speed of each belt and the center of pressure (COP) were sampled at 500 Hz and recorded using Gaitfors^®^software. The system identifies multiple gait events during the walking cycle, including initial contact, toe off and mid stance for each leg. The coordinates of the split belt movement were defined as vertical movement in the *z* direction, anterior–posterior movement in the *y* direction and lateral movement in the *x* direction (**Figure 1A**).

### Data Analysis

Since we were studying motor adaptation, which is thought to be an error-driven process, we adopted the step asymmetry as our measure of the motor error in each cycle (**Figure 1B**):

(1) $$\text{Step asymmetry=}\frac{\text{Left COP length-Right COP length}}{\text{Left COP length+Right COP length}}$$

The COP length was calculated as the distance between the point where the foot touched the belt and the toe of the opposite leg lifted off the belt. When step asymmetry value is zero it describes symmetric walking and when it is other than zero, it means that the gait is not symmetric (Reisman et al., 2005). The underlying assumption of the locomotor adaptation task is that the deviation of the step length from zero is proportional to the error signal that subjects perceive and try to reduce through the adaptation process (Reisman et al., 2009; Torres-Oviedo et al., 2011).

In each test session, we quantified *baseline* error as the mean step asymmetry of the last 50 strides in the baseline period. We define a stride as one gait cycle that started at slow-belt leg initial contact and terminated at the subsequent initial contact of same slow-belt leg. *Early adaptation* was quantified as the mean of the first 10 strides in the adaptation phase, *mid adaptation* error was quantified as the mean of the strides 11–60 in the adaptation phase, *late adaptation* error was quantified as the mean of the motor errors in the last 10 strides of adaptation phase and *after-effect* was quantified as the difference between the mean error of the first 10 strides in the washout phase and the error in late baseline. Mid-errors represents a common model-free approximation for learning rates in locomotor adaptation. *Savings*, faster relearning, was defined as the increase in the learning rate (i.e., change on mid-errors) and *asymptotic performance* was related to the errors during late adaptation. Our definitions of the different errors are in agreement with previous works that investigated locomotor adaptation (Malone et al., 2011; Mawase et al., 2014; Roemmich and Bastian, 2015).

Variability in step asymmetry was defined as the mean squared error (MSE) of the double-exponential function that best fit the individual subject’s data. To demonstrate that our variability estimate stems from execution variability (motor output variability), and not from a slow drifting of step asymmetry values, we calculated the autocorrelation of the residuals of the model (van Beers et al., 2013; Manley et al., 2014).

### Statistical Analysis

Statistical analysis of the data was performed using Matlab with Statistics Toolbox (The MathWorks Inc., Natick, MA, USA) and GraphPad Prism software (The GraphPad Software Inc., La Jolla, CA, USA). Within-group difference in step asymmetry during adaptations between the test sessions (i.e., *T*_1_ and *T*_2_) were assessed with independent two-tailed paired *t*-tests. Differences in after-effect in the washout sessions were assessed using one-way ANOVA. Difference between groups in the first exposure to the perturbation (i.e., *T*_1_) were assessed with two-tailed *t*-test and corrected with Welch’s test if the assumption of equal variances is violated. Welch’s *t*-test is an adaptation of the standard Student’s *t*-test, and is more reliable when the two samples have unequal variances. When the ANOVA results were significant, *post hoc* analysis was conducted using the Holm-Sidak test for multiple comparison. In addition, Pearson correlation test was conducted to examine the relationship between variability and learning rates. To verify that the correlation results were not a result of the small sample, we used bootstrap and permutation tests to estimate the effect of outliers and the null distribution, respectively. Specifically, the bootstrap was performed using 1,000 unique combinations drawn with replacement from the subject pool. The 95% confidence intervals were calculated as the 2.5 and 97.5 percentile values from the distribution for each coefficient obtained across the 1,000 fits. This analysis allows addressing the concern that the correlation that was found was driven by an outlier. In the permutation analysis, correlations were ran on all possible combinations of learning rate and variability changes and a null distribution was computed. Significance level in all comparisons was set at *p* < 0.05.

## Results

### Group Performance during the Adaptation Sessions

**FIGURE 2:**
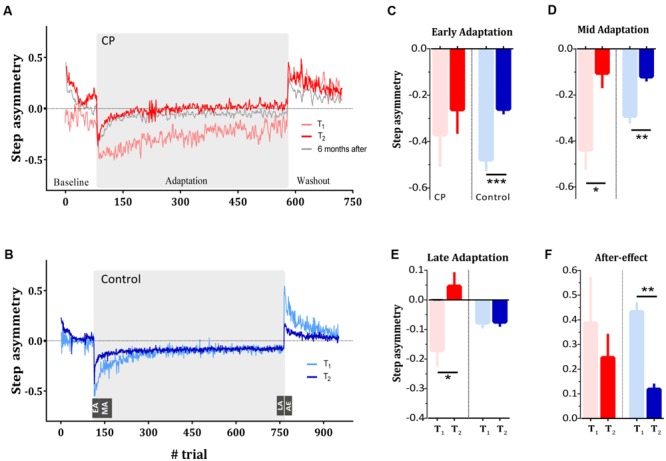
**Long-term learning of a split-belt treadmill in typically developed subjects and subjects with CP. (A)** Step asymmetry (i.e., motor errors) during split-belt adaptation as quantified by the step asymmetry of the subjects with CP during the first (i.e., *T*_1_, light red curve) and last (i.e., *T*_2_, dark red curve) test session. Gray curve represents the adaptation pattern of CP during the 6 months follow up test. **(B)** Step asymmetry of the control group during the first (i.e., *T*_1_, light blue curve) and last (i.e., *T*_2_, dark blue curve) test session. **(C)** Asymmetry in early adaptation (EA) as quantified by the mean of the step asymmetry of the first 10 strides in the adaptation phase. Red bars represent the mean asymmetry of the CP group whereas blue bars represent the mean asymmetry of the control group. In both groups, light bars and dark bars represent asymmetry at *T*_1_ and *T*_2_, respectively. **(D)** Asymmetry in mid adaptation (MA) as quantified by the mean of the step asymmetry of the strides 11–60 in the adaptation phase. **(E)** Asymmetry in late adaptation (LA), representing asymptotic performance, quantified by the mean of the step asymmetry in the last 10 strides of the adaptation phase. **(F)** After-effect (AE) in washout phase of the first and last sessions in both groups. Curves and bars represent the means, and error bars represent the standard error of means (±SE). Significance levels are as follows: ^∗^*p* < 0.05, ^∗∗^*p* < 0.01, ^∗∗∗^*p* < 0.001.

We first sought to test whether subjects with CP show deficits in locomotor adaptation compared to healthy controls. We compared the time course of step asymmetry (i.e., our error estimate) in test sessions conducted before and after 30 and 10 adaptation sessions (*T*_1_ and *T*_2_) in the CP and in the control group, respectively. **Figures 2A,B** show the averaged step asymmetry on a cycle-by-cycle basis for the CP group (**Figure 2A**) and for the control group (**Figure 2B**) in *T*_1_ (light red and light blue curves represent data for the CP and the control group, respectively) and *T*_2_ (dark red and blue curves for CP and control group, respectively). At the beginning of the first adaptation session (*T*_1_), both groups showed a large asymmetric pattern (indicated by the large deviation of step asymmetry from zero). However, while the control subjects gradually reduced asymmetry such that by the end of the session, the asymmetry level got close to baseline level, subjects with CP showed smaller reduction in the asymmetry by the end of the adaptation period. The mean step asymmetry in early adaptation (i.e., computed over the first 10 strides) was -0.38 ± 0.13 (mean ± SE) in the subjects with CP (**Figure 2C**, red light bar) and -0.47 ± 0.05 in control subjects (**Figure 2C**, light blue bar). In the control group, asymmetry levels decreased gradually throughout the adaptation phase, reaching an asymmetry rate of -0.07 ± 0.02 at late adaptation (**Figure 2E**, light blue bar). Subjects with CP exhibited larger asymmetry at late adaptation, reaching levels of -0.16 ± 0.06 (**Figure 2E**, light red bar), suggesting a smaller adaptation to the new perturbation. As expected, in the early washout phase the control subjects showed positive step asymmetry values, representing after-effects [0.43 ± 0.04, one sample two-tailed *t*-test, *t*(8) = 11.05, *p* < 0.001]. Despite the smaller adaptation seen in the CP group, subjects with CP also showed after-effects [mean asymmetry of 0.39 ± 0.18, one sample two-tailed *t*-test, *t*(5) = 2.644, *p* = 0.04].

In sum, in the first exposure to the split-belt perturbation, the CP group showed slower and weaker adaptation (e.g., the light red curve in **Figure 2A**) compared to the control group (e.g., the light blue curve in **Figure 2B**). We further asked if multiple exposures to the same perturbation would show an improvement in learning rate (i.e., savings). We therefore compared the initial performance with the performance of the two experimental groups at *T*_2_, conducted after 30 and 10 sessions of split-belt adaptation training for the CP and control groups, respectively (**Figures 2A,B**).

**Figure 2C** shows the mean asymmetry measure during early adaptation for subjects with CP (red bars) and control subjects (blue bars) across the two test sessions (light color for *T*_1_ and dark color for *T*_2_). We found that while subjects with CP showed comparable error levels in the initial phase of both sessions [*t*(5) = 1.03, *p* = 0.35], control subjects significantly reduced their initial errors in *T*_2_ [*t*(8) = 6.068, *p* < 0.001]. However, examination of the asymmetry measure at mid adaptation show that both groups significantly reduced their mid-errors [*t*(5) = 2.98, *p* = 0.03 and *t*(8) = 4.933, *p* = 0.001 for CP and control groups, respectively] (**Figure 2D**). Therefore, despite the smaller initial adaptation in the CP group compared to control group, both groups showed improvement across sessions (i.e., savings). Of note, savings is typically probed after washing out the adaptation (i.e., after the behavior had returned to a baseline levels). Indeed, our savings probes (i.e., *T*_2_) has been tested after 5 min of tied-belt condition. This baseline period was considered as a washout phase, and the subsequent re-adaptation test was almost free from residuals of previously adapted state (Krakauer et al., 2005; Huberdeau et al., 2015a). We found that CP and control group reached baseline step asymmetry levels (quantified as the mean of the last 50 strides in baseline period) in *T*_2_ that were not different from zero [one sample two-tailed *t*-test *t*(5) = 1.2, *p* = 0.26 and *t*(8) = 1.35, *p* = 0.21 for the CP and control group, respectively]. Interestingly, the retention of the adapted locomotor pattern was also seen in the CP group 6 months after the end of the adaptation sessions (gray curve in **Figure 2A**).

Differences between groups were apparent, however, when examining the changes in asymptotic performance (e.g., the last part of the adaptation phase). ANOVA on the asymmetry at late adaptation revealed a main effect of the test session (*F*_1,13_ = 10.68, *p* < 0.01) as well as a session × group interaction (*F*_1,13_ = 10.07, *p* < 0.01). Within-group comparison results showed that subjects with CP improved their asymptotic performance [*t*(5) = 2.38, *p* = 0.036] whereas control subjects did not [*t*(8) = 0.1409, *p* > 0.89]. These results suggest that training led to a change in the asymptote of the CP group and not of the control group (**Figure 2E**). While the difference in asymptotic levels can be a result of the different number of training sessions that each group performed, the comparable improvement in learning (quantified by mid adaptation step asymmetry), and the highly stable asymptote of the control group before and after training point to a qualitative difference between the groups.

During the training session subjects were exposed only to the perturbation, whereas following the adaptation in the probes washout sessions (which include sessions *T*_1_ and *T*_2_), subjects did a washout session after the adaptation. We were interested in examining whether the improvement in adaptation could also be seen in the washout performance of the subjects. We quantified after-effect as the difference between the mean step asymmetry in the first 10 strides of the washout phase and the mean error in late baseline. This measure therefore represents the process of adapting to the errors caused by the removal of the perturbation. Analysis of after-effect revealed no significant change across sessions in the CP group [*t*(5) = 0.77, *p* = 0.47] but a significant change for the control group *t*(8) = 4.99, *p* < 0.01. Nevertheless, both groups still showed positive step asymmetry during the washout in *T*_2_. Mean step asymmetry was 0.26 ± 0.07 and 0.11 ± 0.02 in subjects with CP and control subjects, respectively (**Figure 2F**). Altogether, this result suggests that the improvements that the subjects with CP showed in the adaptation phase are specific to the learned perturbation, and could not be seen in washouts that was introduced only four times during training.

Another marker of retention that can be seen within each group relate to the step asymmetric pattern at early baseline (i.e., first 10 strides) at the beginning of *T*_2_. This baseline session was preceded by an adaptation epoch that subjects performed on their previous visit to the lab. A large bias at the beginning of *T*_2_ indicate that subjects retained the adapted pattern when exposed to the treadmill again. Result showed that subjects of both groups exhibited a large asymmetric pattern at the beginning of baseline in *T*_2_ [*t*(5) = 4.02, *p* = 0.01 and *t*(8) = 4.614, *p* = 0.0017 for subjects with CP and controls, respectively].

### Changes in Execution Variability during Longitudinal Adaptation

**FIGURE 3:**
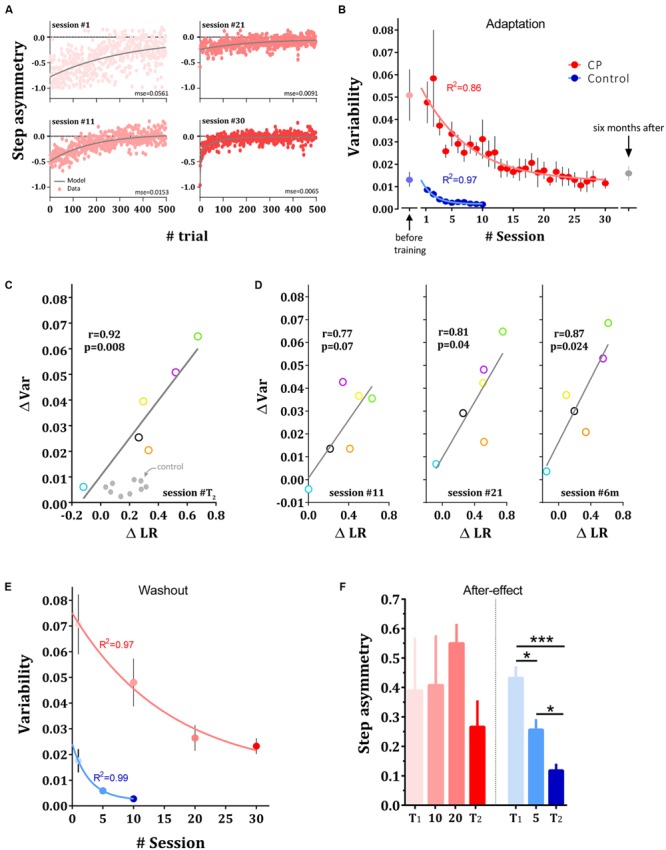
**Changes in variability in adaptation and washout sessions. (A)** Reduction in variability across sessions. Experimental results and model fit. Data points are step asymmetry for representative subject from CP group in sessions 1, 11, 21, and 30. Gray line is the fit for the dual-exponential function. MSE for each session is presented bellow each curve. **(B)** Changes in variability averaged across adaptation sessions in control (blue dots) and CP group (red dots). Faint dots represent the variability during the first baseline session prior to the training. Curves represent the single exponential fit to the mean data for each group, respectively. Gray point represents the execution variability in CP 6 months after the adaptation sessions. **(C)** Inter-subject correlation between the improvements in the estimated learning rate (ΔLR) and the reduction in variability (ΔVar) in CP subjects when comparing *T*_2_ with *T*_1_. **(D)** Correlation between change on learning rate and reduction in variability after 11 sessions (left panel), 21 sessions (middle panel) and 6 months after training (right panel). **(E)** Changes in variance across washout sessions in control (blue dots) and CP group (red dots). Curves represent the single exponential fit to the mean data for each group, respectively. **(F)** After-effect in the washout sessions in both groups. Bars show the mean across subjects and error bars represent standard error of means (±SE). Significance levels is depicted by asterisks –^∗^*p* < 0.05, ^∗∗∗^*p* < 0.001.

In addition to the slower adaptation of the CP group at the beginning of training, their step asymmetry measures were highly unstable and gradually showed improved stability throughout training. To examine the changes in stride-by-stride variability, we measured the changes in variability across sessions. Variability measure during adaptation was defined as the MSE of the double-exponential function that best fit the individual subject’s data (see Materials and Methods). Reduction in execution variability and model fit for sessions 1, 11, 21, and 30 for a representative CP subject is shown in **Figure 3A** and the mean motor variability of CP (in red) and control groups (in blue) over the adaptation sessions are shown in **Figure 3B**. Although subjects with CP showed higher variability than control subjects in *T*_1_ (*p* < 0.0001), their variability decreased significantly across sessions (*p* < 0.01). In addition, to test whether the initial high variability in CP during the adaptation block was not due to increased exploration following the introduction of the perturbation but rather due to a noisy performance, we measured baseline variability prior to the introduction of the perturbation as the variance of the step asymmetry in the last 50 trials in *T*_1_. We found comparable levels of variability to the variability observed during the split-belt perturbation (**Figure 3B**), indicating that the source of variability following the introduction of the perturbation cannot be attributed to exploration (Wu et al., 2014). To ensure that our variability measure was unbiased by the model that we chose, we also quantified variability during adaptation phase using a MSE of the single-exponential fit. The results were consistent (*p* < 0.01).

To further test the evolution of variability reduction, we fit a single exponential function [i.e., y(n) = a ⋅ e^-n/τ^ + c] to the mean variability across subjects. Results revealed a significant (*F*_3,33_ = 96.76, *p* < 0.0001) different curve fit for each data set (*R*^2^ = 0.97 for control group and *R*^2^ = 0.86 for CP group, curves are shown in **Figure 3B**). On average, subjects with CP reduced their variability more slowly than the control group. The time constant of the exponential function of CP (τ = 9.02 [5.9 - 18.31 CI]) was significantly slower than the time constant of the control group (τ = 2.11 [1.5 - 3.56 CI]). Overall, our result suggests that the CP and control groups exhibited a different amount of stride-by-stride unexplained variability and a different rate of reduction of variable errors during adaptation. Subjects with CP showed high variability and a gradual reduction of variability whereas the control group began the experiment with reduced variability and showed an additional, rapid reduction in variability following split-belt adaptation. Although increasing walking speed might influence variability in step length (Hausdorff et al., 2001a,b; Dingwell and Marin, 2006; Dingwell et al., 2007), our results did not support this possibility. We found no correlation between treadmill speed and variability in step asymmetry measured during baseline blocks of the testing sessions *T*_1_ and *T*_2_ (*p* < 0.26).

To validate our conjecture that the step asymmetry variability measurement represents execution noise, and not a slow drifting of step asymmetry values, we calculate the autocorrelation of the residuals for each subject in each group over the test sessions and focused on the first term of this measure (i.e., lag-1 in the autocorrelation; van Beers et al., 2013; Manley et al., 2014). We found that, on average, the first term of the autocorrelation was 0.065 ± 0.04 (mean ± SE) and 0.018 ± 0.03 for control subjects in T1 and T2, respectively, and 0.017 ± 0.037 and -0.056 ± 0.032 for subjects with CP in T1 and T2, respectively. These values do not differ significantly from zero (*p* > 0.13), suggesting that indeed subjects with CP continuously corrected the errors that were captured by the step asymmetry variability measure.

We next enquired whether there is a link between the reduction in variability and the improved learning rates observed following training. In **Figure 3C**, changes in motor variability are plotted against the changes learning rates (normalized mid-errors during adaptation sessions, see Materials and Methods) across subjects with CP. Our result revealed a significant inter-subject correlation between the improvements in learning rate and the reduction in variability (*r* = 0.93, *p* < 0.009, **Figure 3C**), suggesting that the two observations are highly related. Results show that the greater the variability is reduced, the greater the change in learning rate. We next examined whether this pattern of correlation can be also seen at other time points during and after training. We therefore ran correlation analyses comparing performance in *T*_1_ to performance in sessions 11, 21 and 6 months later (**Figure 3D**). We chose days 11 and 21 because subjects in these sessions came to the lab after performing a washout probe session in their former visits (i.e., sessions 10 and 20). These sessions were considered a valid estimate of learning rate since they are not conflated by retention as the case in sessions 10 and 20. The results revealed high correlations between the change in variability measures and change in learning rates at days 21 (*r* = 0.81, *p* = 0.04) and 6 months after training (*r* = 0.87, *p* = 0.024). While the correlation was still high for day 11, the results did not reach a significant level (*r* = 0.77, *p* = 0.07). To verify that the results are not driven by the small sample, we used bootstrap and permutation tests on the main correlation analysis to estimate the effect of outliers and the null distribution, respectively. The bootstrap analysis indicate that the result was not driven by an outlier (*p* = 0.009) and the permutation test demonstrate that the correlation that was found is different from the data-driven null distribution (*p* = 0.01, see Materials and Methods). In contrast to the significant correlations in the group of the subjects with CP, the relationship between variability and learning rate was not apparent in the control group (comparing *T*_1_ and *T*_2_ revealed no significant correlation, *p* > 0.26).

We next examined whether the changes in variability were specific to the adaptation phases (which repeated 30 and 10 times in the CP and control training, respectively) or can also be seen during de-adaptation, a perturbation that was introduced to the subjects only few times throughout training (four times for the CP group and three times for the control group, see Materials and Methods). **Figure 3E** shows the changes in variability in the washout probe sessions for the two groups. Subjects in the two groups showed a marked and significant reduction in variability also in the washout phase (*p* < 0.0001 in both groups).

Interestingly, this generalization of the variability reduction in the CP group to the washout phase is in contrast to the lack of improvement (i.e., reduction) in after-effects (**Figure 3F**). Analysis of after-effects in the washout sessions revealed no significant change across sessions in the CP group (one-way ANOVA, *F*_1.4,7.1_ = 0.81, *p* = 0.44) but a significant change for the control group (one-way ANOVA, *F*_1.6,13.1_ = 19.05, *p* < 0.0001, **Figure 2F**). *Post hoc* comparisons between probe sessions in the control group (between *T*_1_, fifth session and *T*_2_) reveals a continuous significant reduction of step asymmetry during washouts [Holm-Sidak’s test, *t*(8) > 2.9, *p* < 0.017 in all comparisons]. Altogether, this result suggests that the improvements that subjects with CP showed in the adaptation phase are specific to the learned perturbation, and could not be seen for washout, a perturbation that was introduced only four times during training. This result is of particular interest since variability was reduced also during the washout phase.

## Discussion

Individuals with CP suffer from motor impairments from early childhood (Bax et al., 2005; Aisen et al., 2011) that lead to deficits in the ability to adapt to changes in the environments (Gordon and Duff, 1999; Jahnsen et al., 2004; mulMutsaarts et al., 2006; Steenbergen and Gordon, 2006), a limited motor repertoire (Dusing and Harbourne, 2010; Hadders-Algra, 2010) and an increased execution variability (Sanger, 2006; Prosser et al., 2010; Chu et al., 2013; Davies and Kurz, 2013). In the current study, we suggest that adaptation deficits and increased execution variability are related. We report that following prolonged training, subjects with CP show both reduction in variability and improved adaptation suggesting that the initial deficits in adapting to external perturbations might be affected by the increased execution variability in the performance of the people with CP. By exposing subjects with CP to 30 sessions of locomotor adaptation, we were able to find both a reduction in execution variability and an enhancement in the adaptation for the applied perturbation that were highly correlated. During washout sessions, subjects with CP continued to show reduction in the execution variability but they did not show improved ability to adapt.

### Savings in Locomotor Adaptation in Persons with CP

Experimental reports from recent years show that locomotor adaptation, like reaching adaptation, is driven by multiple learning processes that operate at the same time (Malone et al., 2011; Mawase et al., 2014; Roemmich and Bastian, 2015). Savings is thought to be an outcome of a learning process that leads to the generation of long term motor memories. Indeed, it was recently shown that savings in locomotor adaptation involves recall of the experienced errors (Roemmich and Bastian, 2015), similarly to results from reaching adaptation (Herzfeld et al., 2014). On the basis of these results, we argue that the high variability of the subjects with CP affected their ability of adapt during the initial session due to a failure in assigning the source of the perceived error to a stable external perturbation. With training, subjects both reduced their variability, which allowed them to better detect the perturbation, and generated a memory of the adapted pattern. The combination of these effects led to the significant improvement in learning rates following training.

The reasons for the lack of improvement in washout sessions in subjects with CP remain, however, unclear. The fact that reduction in execution variability by itself does not lead to changes in learning rate in washouts suggests that savings in locomotor adaptation cannot be explained by simple Bayesian inference models (Berniker and Kording, 2008; Wei and Körding, 2009) since the reduction in execution variability should have directly affected washout through the reduction in uncertainty regarding the source of the errors. We therefore speculate that our results demonstrate that savings in locomotor adaptation is driven also by previous exposures to the perturbation and reflect a memory of the error (Herzfeld et al., 2014; de Xivry and Lefèvre, 2015; Roemmich and Bastian, 2015) or of a previously reinforced actions (Huang et al., 2011; Huberdeau et al., 2015a,b). CP subjects show savings for the perturbation but not for the washout since they had less exposures to the washout, compared to the amount of exposure to adaptation, and failed to generate a motor memory of that state or to retrieve the required action for that perturbation. Why do the control subjects show savings in washout whereas the CP subjects do not? We can only hypothesize that the higher execution variability in the CP group interfered with the process of generating a long term memory of the context or of the reaction to the perturbation and that additional exposures to the washout would have led to savings in the CP group as well. Difficulties in context-detection during locomotion was also observed in recent study conducted in stroke patients with cerebrum lesions (Reisman et al., 2009). In addition, our observation of slower rate of decay in washouts is consistent with observations in other adaptation experiments that tested cerebellar patients (Xu-Wilson et al., 2009; Criscimagna-Hemminger et al., 2010). Future work will need to further explore context-dependent learning and decay in locomotor adaptation and formally model them (Gershman et al., 2010). Of note, estimation of generalization through washouts should be conducted cautiously since subjects may develop a representation of baseline state during their over ground walking. Nevertheless, the fact that control subjects did show change of after-effects following training, and the large after effects seen in the baseline of *T*_2_, suggest that the representation of the baseline state is task specific. We therefore argue that washout is a valid estimation for a generalization of learning and variability gains to the baseline condition (i.e., no perturbation).

### Increased Execution Variability and Reduced Learning Rates in Persons with CP

The performance of subjects with CP is characterized by deficits in dexterous movements (Duque et al., 2003), increased execution variability (Katz-Leurer et al., 2009; Prosser et al., 2010; Wallard et al., 2012), slower adaptation of predictive forces (Duff and Gordon, 2003; Mawase et al., 2011) and by stereotypical behavior (Hadders-Algra et al., 1999; Hadders-Algra, 2010). These observations are typically explained by different mechanisms, where impairments in dexterous movements are explained by increased execution variability due to damage to the cortico-spinal-tract (Duque et al., 2003), and slower adaptation and increased stereotypical motor performance are explained by limited ability to integrate sensory information of the external environment with the motor output and by a limited motor repertoire. We suggest that increased execution variability leads to impairments in employing compensatory strategies that directly affect the adaptation process. Since the behavior examined here, i.e., walking on a treadmill, is a stereotypical behavior, we cannot directly address a variety of observations pointing to a limited repertoire in performance of persons with CP (Hadders-Algra, 2010). Nevertheless, our results suggest that a limited movement repertoire of CP may be a result of a failure to detect an environmental changes that requires flexibility in action selection. Alternatively, due to the developmental nature of CP, the increased execution variability may have prevented the acquisition of new skills, and led indirectly to an immature motor repertoire. Indeed, locomotor adaptation improves with maturation (Vasudevan et al., 2011) and thus could be linked to developmental processes in the cerebellum or in cerebral regions. The potential effect of immature development on variability and learning is consistent with previous studies that examined the relationship between variability and learning in children (Kuhtz-Buschbeck et al., 1998; Yan et al., 2000; Jansen-Osmann et al., 2002; Yan and Thomas, 2002; Takahashi et al., 2003). These studies showed that performance in children after adaptation is still more variable than in adults and that movement inconsistency due to the immaturity of the motor system, and not a deficit in motor adaptation, ultimately limited their initial motor performance. Furthermore, people with CP also show muscle co-activation and enhanced stretch reflexes, phenomena that can be considered part of the early development of gait and that differ from locomotor patterns in those in whom the cerebral lesion is acquired (Dietz and Berger, 1995; Berger, 1998). While the fact that people with CP improved their adaptation through training suggest that they can learn, we cannot reject the interpretation that the differences between the control subjects and the people with CP stem from the fact that the acquisition of the adapted locomotor pattern in the CP group took longer due to a limited motor repertoire, which is an indirect outcome of their increased execution variability.

Our results suggest a close but negative relationship between variability during execution and learning rate. Subjects that showed larger reduction in variability are more likely to improve their learning rate during walking. A recent study, however, suggests a positive relationship between exploration variability at baseline and learning rate in reach movement (Wu et al., 2014). Although it is possible that our measure of variability contains an exploration component, our results could not be explained by exploration. The fact that variability levels were lower in the control group that showed higher learning rates further support this conjecture. Of a particular interest is the fact that the control subjects did not show a correlation between the reduction in variability and the improvement in learning rates. While this result can be an outcome of the difference in the number of training sessions between the groups, we speculate that it points to a qualitative difference between the experimental groups. We suggest that the reduction in variability in the CP group reflects a reduction of impairment that enabled the improvement in adaptation through an improvement in error assignment. While admittedly the control group also showed reduction in variability and improvement in learning rate, this group did not show correlation between these variables. We speculate that this result indicate that the improvement in learning rate in the control group was not related to the reduction in variability in this group and that both processes improved independently. Alternatively, the lack of correlation could be explained by the limited distributions of the variability and learning rate changes in the control group, possibly due to ceiling effects (**Figure 3C**). Nonetheless, our correlation results could not imply causation between execution variability and leaning rates and they might even reflect an opposite dependency (van Beers, 2009) or dependence on a third unmeasured process. More work is needed to carefully test the causality between learning and execution variability.

### Changes in Asymptotic Performance in Persons with CP

One of the intriguing results is that subjects with CP show changes in step asymmetry levels at asymptote whereas the control subjects did not change their asymptote and maintained stable negative values of step asymmetry. While this result should be taken cautiously due to the difference in the number of sessions between the groups, the stability of asymptote in the control group (**Figure 2E**) is noteworthy. Two possible explanations can account for the apparent difference in the asymptotic levels of the two groups. First, according to the state space approach, asymptote reflects a balance between learning rate (i.e., sensitivity to error) and forgetting rate. The typical incomplete adaptation in the control group, as shown by the negative values of the asymptotic level, can be described as an equilibrium between stride-to-stride forgetting and the amount of learning from the experienced error. This balance means that there is always some forgetting between strides which make the learning incomplete. Our results suggest that the balance between learning and forgetting in the subjects with CP is less stable across sessions compared to the control group. We speculate that the change in the asymptote of the CP group might be a result of an involvement of an additional success-related mechanism that operates in parallel to the dominant error-based process. It was shown that such reinforcement process underlies long-term memory retention (Abe et al., 2011; Shmuelof et al., 2012a; Haith and Krakauer, 2013; Galea et al., 2015).

Second, explicit knowledge about the perturbation may also affect asymptotic performance. A recent study showed that healthy control subjects tend to underestimate the true value of the speeds during split-belt adaptations and thus showed incomplete learning (Roemmich and Bastian, 2015). Despite the observation that diverse training schedules (such as different variations of abrupt vs. gradual perturbation) affect differently recall of the gait pattern in subsequent tests, almost all subjects in all environments underestimate the true speeds. In other words, it is possible that control subjects had errors in estimating the perturbation and subsequently tried to minimize prediction errors which are derived based on their erroneous estimate of the perturbation rather than based on its true values. In this case, the incomplete adaptation of the controls reflects their estimated “complete” adaptation. This perspective raise the idea that the longer training of the subjects with CP, or their increased execution variability, allowed them to acquire a more accurate explicit estimation of the perturbation and to subsequently adapt for it completely.

The interpretation of the differences between the group of the subjects with CP and the control group of the healthy subjects should be done cautiously due to the difference in the number of sessions that the two groups performed (30 locomotor adaptation sessions in the CP group and 10 in the control group) and the timing of the first washout period (after five sessions in the CP group and after 10 sessions in the control group). It should be noted, however, that the main objective of the current study was to explore the relationship between learning abilities and execution variability during a longitudinal training in subjects with CP and not to directly compare their performance with controls. The idea behind running the control group was to qualitatively compare the initial performance of the groups, and the sensitivity of each one of the groups to a longitudinal training protocol in terms of changing in learning rates, asymptotic levels and washout performance. Another limitation of the study is the small sample size. Null results in comparisons within and between groups may be explained by insufficient power and not by lack of effect. Larger longitudinal studies with comparable trainings for the experimental and control groups are needed to facilitate group comparisons and to substantiate some of the speculations that are made here.

### Rehabilitation Aspects and Future Directions

Our results highlight the relationship between execution variability and motor adaptation in subjects with CP during a prolonged practice. The apparent deficit in adaptation of persons with CP seems to be at least partially caused by their increased performance variability rather than by their inability to adjust to new motor behaviors. This study provides evidence for the potential effect of improvement in execution through training on the ability of subjects with CP to learn to act in new environments. Our findings may therefore bear an important insight for the field of rehabilitation, since they emphasize the importance of focusing on the change of motor variability (i.e., second-order moment) closely along the learning rate (i.e., first-order moment) during the process of motor learning and suggest that reduction of execution variability through repetitive and prolonged training protocol may be a precursor for enhanced adaptation and motor skill learning processes. The current study also opens an opportunity to test in future research the causality between variability and learning in different motor tasks. While at this point we cannot argue for a similar effect of execution variability on the acquisition of new abilities (motor skills, Shmuelof et al., 2012b) or on the reacquisition of lost abilities, we predict that increased execution variability would slow down any motor learning. Furthermore, since generalization of the improvement in error attribution is key for the development of effective rehabilitative treatments, it may be that combining other protocols, such as random practice, with a variability reduction treatment, may prove to be more effective (Plautz et al., 2000; Giuffrida et al., 2009).

## Author Contributions

FM, SB-H, AK, and LS conception and design of research. FM, KJ, and LR performed experiments. FM and LR analyzed data. FM, SB-H, and LS interpreted results of experiments. FM prepared figures. FM drafted manuscript. FM, SB-H, and LS edited and revised manuscript. FM, SB-H, and LS approved final version of manuscript.

## Conflict of Interest Statement

The authors declare that the research was conducted in the absence of any commercial or financial relationships that could be construed as a potential conflict of interest.
